# Humeral shaft reconstruction surgery following military combat gunshot injury using a titanium spinal mesh cage - A case report

**DOI:** 10.1016/j.ijscr.2025.111772

**Published:** 2025-08-05

**Authors:** Rohil Singh Kakkar, Rohan Maske, Vinayak Garje, Ananya Pareek

**Affiliations:** a Consultant Orthopaedic Surgeon, Eternal Hospital, Jaipur, India; b Consultant Orthopaedic Surgeon, Noble Hospital, Pune, India; c Consultant Medical Oncologist, RHL Renova Cancer Center, Jaipur, India

**Keywords:** Military trauma, Gunshot fracture, Humerus reconstruction, Spinal cage, Limb salvage, Bone graft

## Abstract

**Introduction:**

High-velocity gunshot injuries to the upper extremity are rare but devastating, particularly in military personnel, and are associated with high risks of infection, instability, and limb loss. Reconstruction of extensive segmental bone defects in such cases is a complex surgical challenge.

**Case presentation:**

We report a challenging case of a 32-year-old Indian Air Force soldier who sustained a Grade 3B open right humeral shaft fracture from a gunshot wound during military combat. Initial management included wound debridement, skin grafting, and external fixation at military hospital. Three months later, he presented to our center with a 10 cm segmental bone defect, gross arm instability, and stiffness in shoulder and elbow joint. Definitive humeral reconstruction was carried out using a fibular graft engrafted into a customized spinal cage and stabilization following a fixation with a plate. Long-term follow-up confirmed fracture union and full functional recovery of the limb.

**Discussion:**

Gunshot injuries pose unique challenges due to bone comminution, soft tissue damage, and instability. Conventional techniques like bone transport or shortening may not provide sufficient anatomical and biomechanical restoration. The spinal mesh cage served as a structural scaffold, reducing the required volume of autograft while ensuring mechanical stability.

**Conclusion:**

Gunshot fractures of the humerus should be approached as a distinct clinical entity. The use of a titanium spinal mesh cage provides anatomical alignment, mechanical stability, and a scaffold for autologous bone grafting, making it a viable option for reconstructing large upper limb defects.

## Highlights

• Military combat injuries demand a multidisciplinary approach for optimal limb salvage.
• First Indian case report of humeral shaft reconstruction utilizing a titanium spinal mesh cage.
• Military combat-related gunshot wounds often result in severe osseous and soft tissue disruption.
• Intramedullary spinal mesh cage provides a stable scaffold for bone regeneration and fixation.

## 1 Introduction

High-energy gunshot injuries to the upper limb, particularly those sustained in military combat, pose significant surgical challenges due to the combined impact of extensive osseous destruction, severe soft tissue trauma, and gross wound contamination (1,2). These injuries frequently result in complex, comminuted open fractures with associated segmental bone loss, necessitating a staged, multidisciplinary approach to optimize outcomes. Among these, Grade 3B open fractures of the humeral shaft are particularly difficult to manage due to extensive periosteal stripping, bone fragmentation, and the high risk of deep infection and non-union (3). Initial management typically includes thorough debridement, provisional stabilization with external fixation, and soft tissue coverage. Definitive reconstruction is delayed until the wound bed is clean and stable (4). Although external fixation serves as an effective temporary stabilization tool, the reconstruction of large diaphyseal bone defects remains a major orthopaedic challenge, especially when the defect exceeds 6 cm (5). Conventional methods such as autologous bone grafting, Ilizarov bone transport, and iliac crest bone grafts have shown variable success rates but are often hindered by prolonged treatment duration, donor site morbidity, and significant technical demands (6). Titanium mesh cages, originally designed for vertebral body reconstruction in spinal surgery, have more recently been explored for long bone reconstruction. These cages offer a dual advantage: they provide immediate mechanical stability and serve as a scaffold for bone graft incorporation (7,8). However, their use in humeral shaft reconstruction, particularly following ballistic trauma, remains sparsely reported in the literature (5,7). In this report, we describe the successful reconstruction of a 10 cm segmental midshaft humeral defect in a young Indian Air Force soldier using a customized spinal mesh cage filled with fibular autograft. To the best of our knowledge, this represents the first reported case in India employing this novel technique for upper limb salvage in a post-gunshot scenario. This case report has been reported in line with the SCARE checklist (9).

## 2 Case presentation

**Fig. 1:**
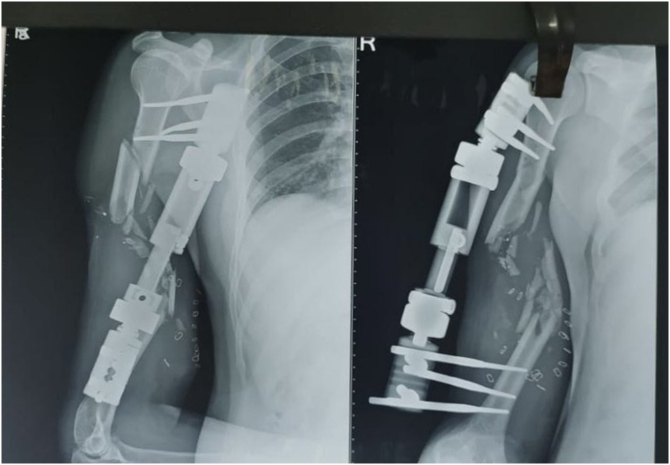
X-ray of the right humeral shaft anteroposterior and lateral views illustrate damage control procedure primarily performed by the military surgeon through fracture stabilization with an external fixator.

**Fig. 2:**
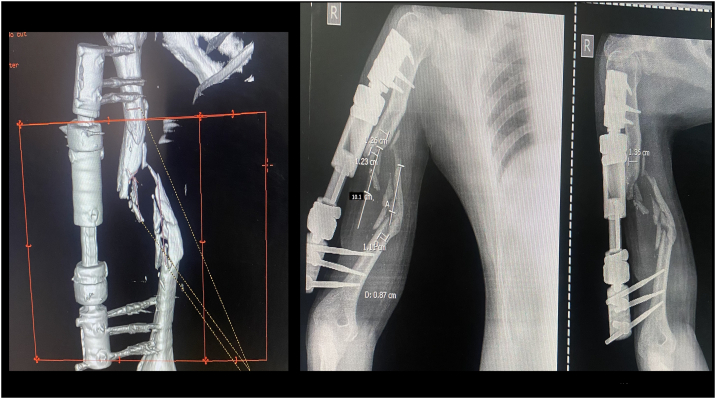
Patient presented to us three months post initial procedure, CT scan and X-rays of right arm depicted a 10 cm segmental defect of the humeral diaphysis with an external fixator in-situ.

**Fig. 3:**
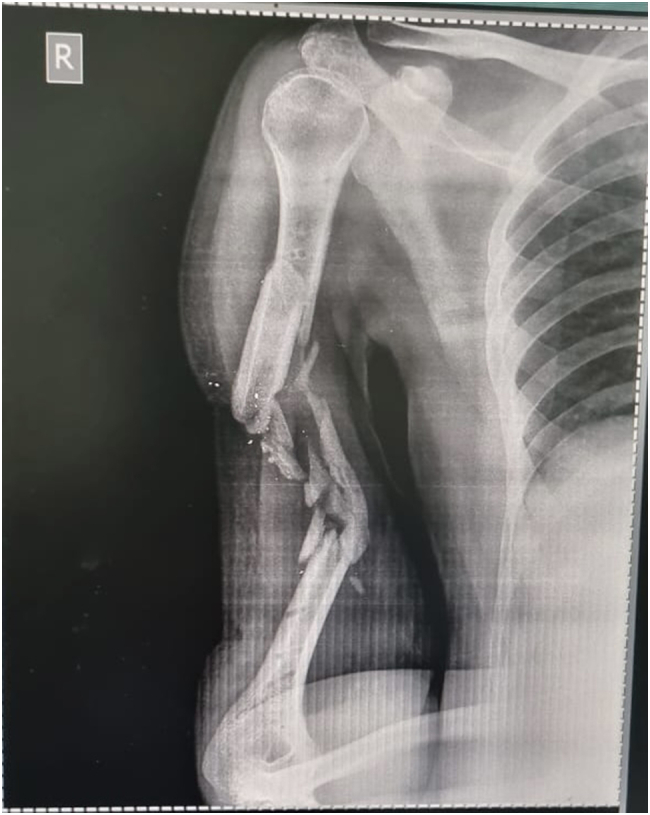
X-ray of the right humerus anteroposterior (AP) view after removal of external fixator before final fixation.

**Fig. 4:**
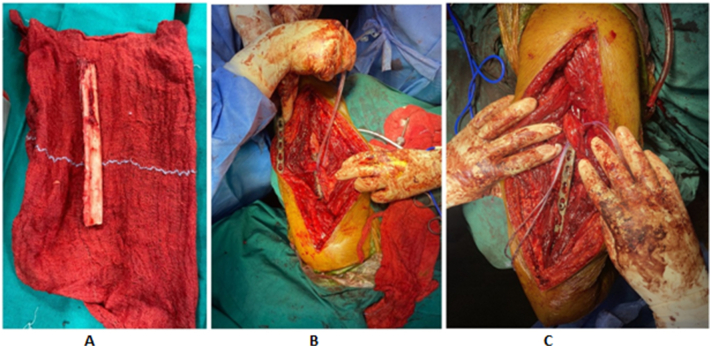
a-c: Intra-operative images of right humerus reconstruction following segmental bone loss. (Fig. 4a: Illustrates harvested fibular graft prepared for reconstruction, Fig. 4B: Illustrates fibular graft placed within a titanium spinal mesh cage and inserted into the humeral shaft defect in a press fit manner, and Fig. 4C: Illustrates final fixation with long locking compression plate and screws applied to the posterior cortex securing the cage-graft construct.

**Fig. 5:**
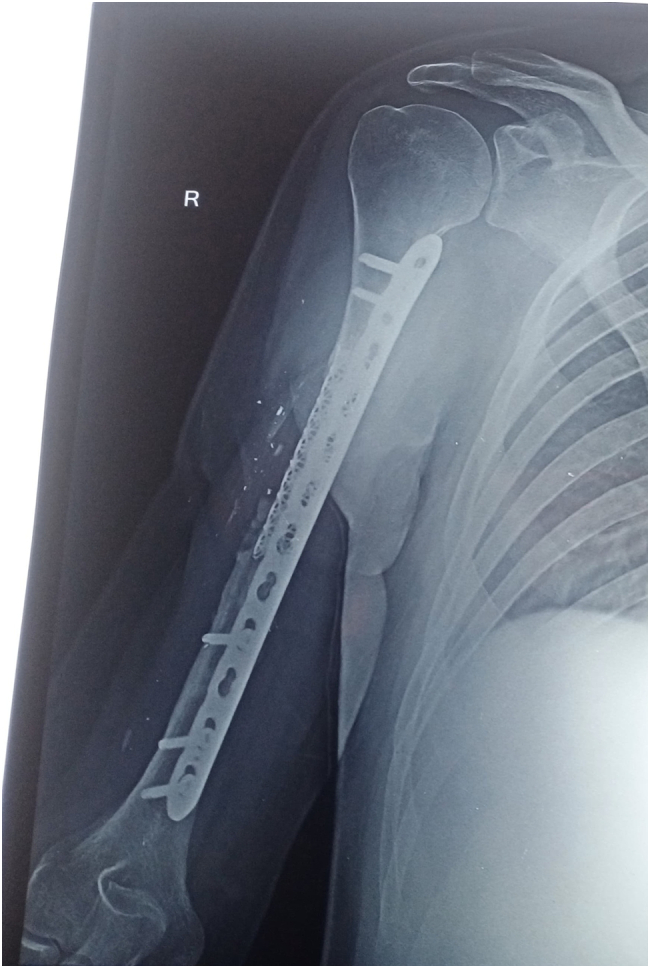
Immediate postoperative xray of the right arm illustrates humeral mid-shaft reconstruction utilizing a customized spinal mesh cage engrafted with fibular graft and stabilized with a long plate and screws.

**Fig. 6:**
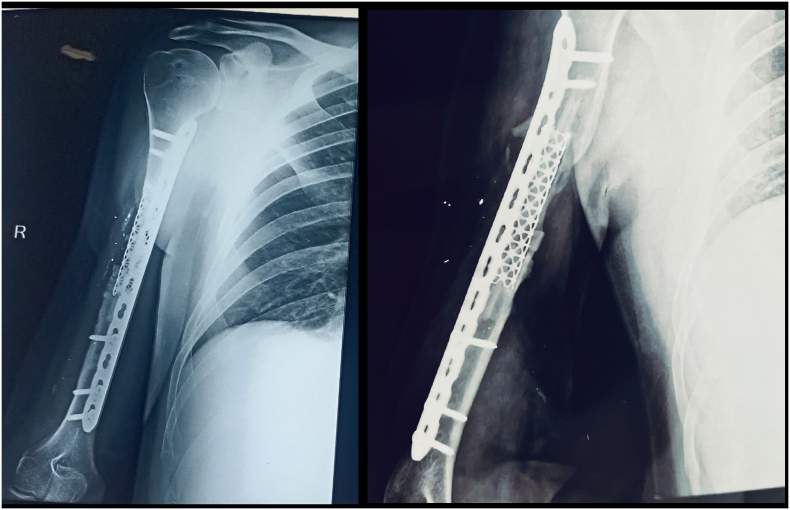
Anteroposterior (AP) and lateral radiographs of the right arm at 2-year postoperative follow-up depicts integration of bone graft with solid union around the cage.

**Fig. 7:**
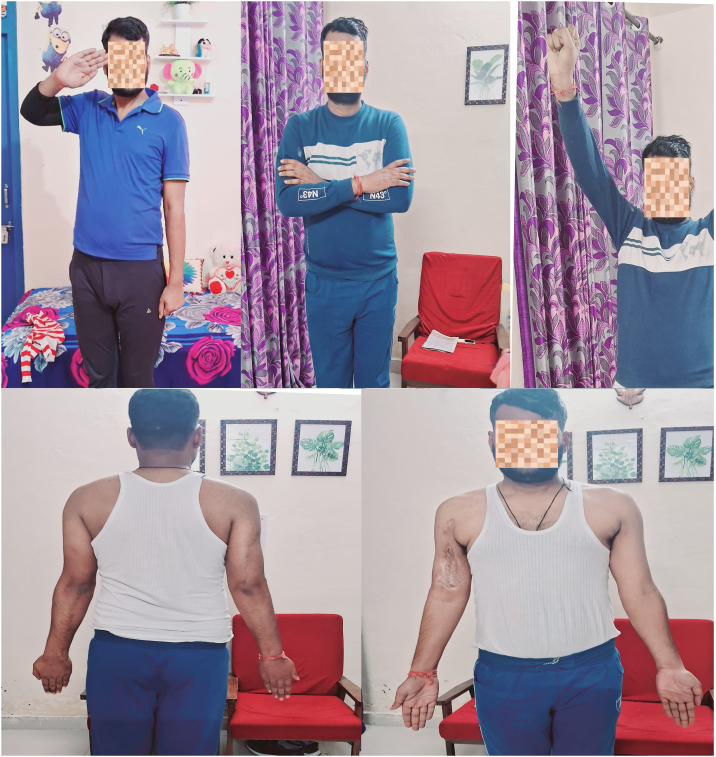
At 2-year follow-up, radiological and clinical union was achieved, restoring full arm function and enabling the patient's return to military service.

A 32-year-old right-handed male soldier from the Indian Air Force sustained a high-velocity gunshot injury to the right upper arm during military combat. The projectile caused a large open wound measuring approximately 17 cm × 11 cm × 4 cm over the anterior mid-arm region. Clinical examination revealed no neurovascular deficits in the affected limb. Radio-clinical evaluation depicted a Grade 3B open comminuted midshaft fracture of the right humerus with a segmental bone defect estimated at 10 cm in length. The patient had no significant past medical history, was a non-smoker, and had no known comorbidities. Initial management at a primary military hospital involved thorough wound debridement, soft tissue coverage with a split-thickness skin graft, and skeletal stabilization using a unilateral rail-type external fixator (Fig. 1). Due to the extent of the bone loss and soft tissue fibrosis, he was offered an arm-shortening procedure for limb salvage. However, he declined this intervention and presented three months later to our facility - Marble City Hospital, Kishangarh for further reconstructive options. At presentation, the skin graft had healed well, but the right arm was grossly unstable, and both the shoulder and elbow joints were completely stiff and non-functional. Radiographs and CT imaging confirmed a persistent 10 cm midshaft bone defect with the external fixator in situ (Fig. 2). A detailed preoperative plan was formulated using patient-specific imaging to determine the appropriate reconstructive strategy. Two weeks prior to definitive surgery, the external fixator was removed under aseptic precautions and prophylactic intravenous antibiotics were administered to reduce infection risk (Fig. 3). Definitive reconstruction was performed under general anesthesia in the lateral decubitus position. A 12 cm segment of ipsilateral fibula was harvested using a standard lateral approach. Through a posterior approach to the humerus, the radial nerve was identified, isolated, and protected using a vessel loop. Intraoperatively, the fracture site was found to be fibrotic and multi-fragmentary. All necrotic bone fragments were excised until bleeding viable bone ends were obtained, confirming a final segmental defect of approximately 10 cm. To reconstruct the defect, a titanium spinal mesh cage (Medtronic®, USA) with dimensions 12 mm in diameter and 110 mm in length, originally designed for vertebral reconstruction was selected based on preoperative templating. The cage was manually adjusted to fit the medullary canal and was packed with the harvested fibular graft. This construct was then press-fitted into the humeral canal to restore anatomical alignment. Mechanical stabilization was achieved using a 4.5 mm narrow locking compression plate (DePuy Synthes®, Switzerland) applied to the posterior cortex. The proximal screws were inserted through the fibular graft and mesh cage to enhance construct stability and prevent toggling (Fig. 4, Fig. 5). Standard layered wound closure was performed over a suction drain. Supervised physiotherapy was initiated on postoperative day one to encourage shoulder and elbow mobility. Sutures were removed on postoperative day 16. At the 2-year follow-up, the patient demonstrated radiographic fracture union with a pain-free, functional range of motion of the shoulder and elbow, and no residual instability (Fig. 6, Fig. 7).

## 3 Discussion

High-velocity gunshot injuries, particularly in military combat settings, produce severe musculoskeletal damage due to the immense kinetic energy transferred to both osseous and soft tissues. These injuries are frequently accompanied by gross contamination and bone comminution, necessitating thorough debridement and staged management using external fixation under the damage control orthopaedics protocol (1,2). Rail or ring fixators provide provisional stabilization, maintain limb length, and allow soft tissue healing while minimizing infection risk in the acute phase. When faced with massive segmental bone loss in the humerus, definitive reconstruction remains a major surgical challenge. Several techniques have been described in the literature, each with specific biomechanical and biological considerations. The vascularized fibular graft (VFG) is considered a gold standard for segmental reconstruction due to its osteogenic, osteoinductive, and osteoconductive potential (3). However, it requires microsurgical expertise, prolonged operative time, and carries donor site morbidity, making it less feasible in resource-limited settings or urgent trauma scenarios (4). The Masquelet technique, involving a two-stage induced membrane procedure followed by bone grafting, has demonstrated promising results in lower limb reconstructions, but upper-limb applications remain limited, particularly when membrane formation is inadequate or delayed (5,6). Ilizarov bone transport is another widely used option, especially for lower extremities, but presents drawbacks in the upper limb: extended treatment duration, high demands on patient compliance, and complications such as regenerate insufficiency, pin tract infections, and joint stiffness (7). Segmental allografts, while providing immediate structural support, carry risks of immunogenic rejection, graft resorption, and fatigue failure, and they require access to bone banks and strict infection protocols often unavailable in combat zones or rural India (8). Intercalary endoprostheses, more commonly used in oncological reconstructions, provide immediate functional restoration but lack biological incorporation, and are prone to mechanical failure in younger, high-demand patients such as active military personnel (9). In this context, our hybrid strategy using a titanium spinal mesh cage combined with a non-vascularized autologous fibular graft represents a pragmatic and biomechanically stable alternative. The mesh cage acted not only as a scaffold for fibular incorporation but also as a mechanical containment device, ensuring proper alignment and load-sharing. The construct was further stabilized with a posteriorly applied 4.5 mm narrow locking compression plate, allowing immediate alignment restoration and early mobilization. While non-vascularized fibular grafts are technically simpler and widely used for humeral reconstructions, their primary limitation is the risk of late complications—particularly nonunion, resorption, or fatigue fracture due to compromised blood supply (10,11). Studies have reported nonunion rates of up to 20 % in such grafts used for defects >6–8 cm, especially when stabilization or graft-host integration is suboptimal ([12], [13], [14], [15]). However, recent biomechanical analyses suggest that intramedullary support, as provided by mesh cages, can enhance load distribution and reduce micromotion, potentially mitigating these risks (16). A 2023 systematic review by Sharma et al. (17) analyzed 42 cases of post-traumatic humeral diaphyseal defects and concluded that dual-mechanism constructs - combining structural scaffolding with biologic bone demonstrated superior union rates compared to traditional autografting or distraction osteogenesis alone. Similarly, Zhang et al. (18) reported that custom implants (including 3D-printed cages and mesh sleeves) offer improved early stability, reduced complication rates, and faster rehabilitation in long bone defects. To the best of our knowledge, this is the first documented Indian case utilizing a custom titanium spinal mesh cage for post-gunshot humeral shaft reconstruction. Our technique demonstrates how resource-adapted innovations can bridge the gap between biological healing and mechanical support in limb-salvage surgery, particularly in trauma scenarios where conventional techniques are either unavailable or unsuitable.

## 4 Conclusion

Gunshot-related humeral fractures require specialized consideration distinct from conventional open fractures. Use of a spinal mesh cage serves as a structural scaffold, enabling reduced graft requirement while ensuring both anatomical alignment and mechanical stability. This technique offers a viable salvage option for extensive upper limb defects, especially in high-stakes military trauma.

## Author contribution

Rohil Singh Kakkar: Data curation, writing - original draft preparation, supervision, reviewing and editing

Rohan Maske: Conceptualization, methodology

Vinayak Garje: Visualization, Investigation

Ananya Pareek : Investigation

## Consent

Written informed consent was obtained from the patient for publication of this case report and accompanying images. A copy of the written consent is available for review by the Editor-in-Chief of this journal on request.

## Ethical approval

This is a case report. Therefore, it didn't require ethical approval from the ethics committee of Marble City Hospital, Kishangarh, Rajasthan. This manuscript has not been published elsewhere.

## Guarantor

Dr Rohil Singh Kakkar, MS Ortho accepts full responsibility for the integrity of the content presented in this article. As the guarantor, Dr Rohil Singh Kakkar affirms that the research, data, and conclusions presented are accurate, complete, and represent the work performed. The guarantor has overseen the project to ensure that all ethical guidelines and research standards were followed and that all co-authors have approved the final version of the manuscript.

## Funding

No external funding was received for this study.

## Declaration of competing interest

The authors declare no conflicts of interest.
